# Outbreak of carbapenem-resistant *Acinetobacter baumannii* carrying the carbapenemase OXA-23 in ICU of the eastern Heilongjiang Province, China

**DOI:** 10.1186/s12879-019-4073-5

**Published:** 2019-05-22

**Authors:** Yongxin Zhao, Kewang Hu, Jisheng Zhang, Yuhang Guo, Xuecai Fan, Yong Wang, Sedzro Divine Mensal, Xiaoli Zhang

**Affiliations:** 1 Department of Microbiology, First Affiliated Hospital of Jiamusi University, Jiamusi, Heilongjiang Province China; 2 Second Affiliated Hospital of Jiamusi University, Jiamusi, Heilongjiang China; 3 Jiamusi University, Jiamusi, Heilongjiang China

**Keywords:** *Acinetobacter baumannii*, Carbapenem resistant, Outbreak, Infection control, OXA-23, CC2

## Abstract

**Background:**

To investigate the carbapenem resistance mechanisms and clonal relationship of carbapenem-resistant *Acinetobacter baumannii* (CRAB) strains isolated in the intensive care unit (ICU) of the First Affiliated Hospital of Jiamusi University, management approaches to ICU clonal CRAB outbreaks were described.

**Methods:**

The sensitivity of the antibiotic was determined using the VITEK-2 automated system. Carbapenemase genes (*bla*_TEM_, *bla*_SHV_, *bla*_KPC_, *bla*_NDM_, *bla*_IMP-4_, *bla*_VIM_, *bla*_OXA-23_, *bla*_OXA-24_, *bla*_OXA-51_, and *bla*_OXA-58_), *AmpC* enzyme genes (*bla*_ACC_, *bla*_DHA_, *bla*_ADC_), and *ISAba1* were assessed for all collected isolates using polymerase chain reaction (PCR). The transfer of resistance genes was investigated via conjugation experiments. The clonal relationship of isolates was determined via enterobacterial repetitive intergenic consensus (ERIC)-PCR and multilocus sequence typing (MLST). When the detection rate of CRAB increased from 25% in 2010 to 92% in 2014, a number of actions were initiated, including enhanced infection control, staff education, and the cleaning of the hospital environment.

**Results:**

Clinical isolates were positive for the following genes: *bla*_OXA23_, *bla*_OXA51_, *bla*_OXA24_, *bla*_ADC_, *bla*_TEM_, *ISAba1*, *ISA-23*, and *ISA-ADC*; however, *bla*_OXA58_, *ISA-51*, *bla*_NDM_, *bla*_IMP_, *bla*_KPC_, *bla*_TEM_, *bla*_SHV_, *bla*_VIM_, and *bla*_ACC_ were not detected. Four carbapenem-resistant isolates successfully transferred plasmids from *A. baumannii* isolates to *E. coli* J53. MLST showed that all strains belonged to ST2 except for one isolate, which belonged to the new genotype ST1199. The ERIC-PCR method found the following three genotypes: type A in 8, type B in 12, type C in 1, and two profiles (A, B) belonged to ST2. After taking control measures, the prevalence of CRAB isolates decreased, and the discovery rate of CRAB dropped to 11.4% in 2017.

**Conclusion:**

The obtained result suggests that *bla*_OXA-23_-producing CC2 isolates were prevalent in the ICU of the First Affiliated Hospital of Jiamusi University. Targeted surveillance was implemented to identify the current situation of the ICU and the further implementation of infection control effectively prevented the spread of nosocomial infection.

## Background

*Acinetobacter baumannii (Ab)* is a Gram-negative pathogen that can cause opportunistic infection, ventilator-related pneumonia, as well as infections of the bloodstream, urinary tract, skin, and soft tissue [1, 2]. It has been widely reported in health-care settings and hospitals worldwide. In particular, patients with low immune function and hospitalized in intensive care units (ICU) are more likely to be colonized or infected with *A. baumannii*.

Carbapenems have attracted wide attention due to their high antimicrobial activity and low toxicity. However, *A. baumannii* has developed resistance to this drug, resulting in a major global health concern. It has been reported that about 65% of *A. baumannii* pneumonia in the United States and Europe was caused by carbapenem-resistant *A. baumannii* (CRAB). Similarly, its infection rate in China has reached 62% or more [3]. CRAB is primarily associated with the production of class D carbapenem-hydrolyzing-lactamases (CHDL) and less frequently with metallo-*β*-lactamases (MBL) [4]. In particular, there are six subgroups associated with *A. baumannii* resistance: OXA-51-like, OXA-23-like, OXA-40-like, OXA-58-like, OXA-143-like, and OXA-235-like [5]. Previously, Liu et al. reported the dissemination of multidrug resistant (MDR) *bla*_OXA-23_-producing *A. baumannii* clones in 18 provinces of China [6]; however, there is a lack of knowledge about the molecular mechanism of CRAB in Heilongjiang province, China.

The MLST has been extensively utilized for genotyping of bacteria. Molecular epidemiological studies of *A. baumannii* have shown that CC2 has engaged a crucial role in nosocomial infection outbreak and spread in China [7]. The current report focuses on investigating the carbapenem resistance mechanisms and the molecular epidemiology of CRAB in ICU of eastern China. Furthermore, the control measures of an outbreak of CRAB were described in the ICU.

## Methods

### Clinical isolates and antimicrobial susceptibility testing (AST)

The outbreak occurred at the First Affiliated Hospital of Jiamusi University (a 1800-bed hospital in Heilongjiang Province, China). Twenty-one non-repeated clinical CRAB strains were collected from the ICU. The ICU consists of six wards; different wards are separated from each other, but there is no separate isolation room. The specific ICU has 60 beds and can provide care for critically ill patients in the same hospital or in other hospitals. Data was collected from September 2013 to January 2015 (Due to improper preservation of the strain, two strains died; therefore, these were excluded from the experimental data), and all isolates were frozen until further use. Carbapenemase-producing isolates were screened by the Modified Hodgge test (MHT) and the results were interpreted according to CLSI2016. The clinical characteristics of 21 patients were also reviewed, and several characteristics were assessed including sample type, presence of indwelling catheters or other devices, duration of hospital stay, and outcomes. Species identification and minimum inhibitory concentrations (MICs) of all tested antibiotics were analyzed using the Vitek 2 Compact system (BioMerieux, France). The identity of 21 isolates was further confirmed using 16S rRNA gene sequences and reaction conditions as previously described [8]. Sequencing positive amplification products and the results were compared against the BLAST tool.

### Molecular detection of resistance genes

DNA was extracted using the boiling method. Carbapenemase genes (*bla*_TEM_, *bla*_SHV_, *bl*a_KPC_, *bla*_NDM_, *bla*_IMP-4_, *bla*_VIM_, *bla*_OXA-23_, *bla*_OXA-24_, *bla*_OXA-51_, and *bla*_OXA-58_) and *AmpC* enzyme genes *(bla*_ACC_, *bla*_DHA_, and *bla*_ADC_) were assessed for all collected isolates using PCR [9–11]. PCR amplification was performed using the insertion sequence ISAba1 with the alleles *bla*_OXA-51_, *bla*_OXA-23_, and *bla*_ADC_, respectively, to assess the resistance to carbapenemases [12]. The test data were analyzed using Bioedit software, and the results were compared by online blast software.

### Conjugation experiments

As previously described we conducted the conjugation experiment using a membrane bonding method [13]. CRAB and *E. coli* J53 were mixed as donor and recipient bacteria on Luria-Bertani agar at a ratio of 1:4, respectively, and the mixtures were incubated overnight at 37 °C. Transconjugants were selected from a mixed broth containing sodium azide and imipenem. The concentrations of sodium azide and imipenem in LB broth were 100 μg/ml and 1 μg/ml, respectively. The VITEK 2 compact system identified colonies that grew on the selective medium. The presence of transconjugant resistance genes was determined by PCR and nucleotide sequencing techniques.

### Enterobacterial repetitive intergenic consensus-PCR (ERIC-PCR) and multilocus sequence typing (MLST)

Genetic relatedness of isolates was determined by ERIC-PCR and MLST. The primer ERIC-2 5′-AAGTAAGTGACTGGGGTGAGCG-3′ was used for amplification. PCR conditions were described previously [14]. The ERIC-PCR fingerprint of *A. baumannii* was analyzed using Tocan Gel analysis software (Shanghai, China). Cluster analysis was performed using NTSYS-pc software according to the method reported by Dos Anjos et al. Code 1 indicates the presence of a polypeptide fragment and code 0 indicates the absence of the polypeptide fragment. On the basis of Dice’s similarity coefficient(1% position tolerance) and the unweighted pair group method arithmetic averages (UPGMA) were used, and similarity values greater than 90% classified as the same genotype, representing the same clonal type. In addition, each isolate was regarded as an operational taxonomic unit (OTU) [15]. Seven housekeeping genes including cpn60, fusA, gltA, pyrG, recA, rplB, and rpoB were amplified and sequenced; then, the sequencing results were aligned on the Pasteur Institute’s MLST website (www.pasteur.fr/mlst). The eBURST algorithm (version 3; http://eburst.mlst.net/) was used to assign clonal complexes (CCs).

### Outbreak control measures

In February 2015, an outbreak control team was organized to control the further spread of CRAB. The following measures were implemented to strengthen infection control: (1) Cohorting of patients in designated areas of the ICU. New admissions were separated from infected or settled patients and were cared for by different medical groups. (2) Strengthen the hand hygiene awareness of medical staff and take strict preventive measures to control the spreading of CRAB, such as placing an alcohol hand disinfectant in the room. (3) After patient discharge, reduce environmental pollution by cleaning all domains of the ward besides equipment. (4) Changes in antibiotic policy: colistin and carbapenems could only be used after approval during the outbreak.

## Results

### Results of outbreak control measures

From 2010 to 2017, 222 strains of *A. baumannii* were detected in the hospital ICU. The ICU detection rate of *A. baumannii* increased from 19% in 2010 to 34.4% in 2017, and the detection rate of CRAB increased from 25% in 2010 to 92% in 2014. Of the 25 unrepeated sequences in 2014, 23 were CRAB strains. However, since the execution of infection control measures in 2015, the discovery rate of CRAB has begun to decline, and the discovery rate of CRAB in 2017 dropped to 11.4%, as shown in Table 1.

**Table 1:** Detection of CRAB (%) in ICU patients during the year 2010 to 2017

Year	Total number of isolates in the ICU	Acinetobacter baumannii		CRAB	
		counts	detection rate^a^%	counts	detection rate^b^%
2010	42	8	19.0	2	25.0
2011	48	5	10.4	4	80.0
2012	41	8	19.5	7	87.5
2013	65	12	18.5	10	83.3
2014	87	25	28.7	23	92.0
2015	117	56	47.9	18	32.1
2016	172	29	16.9	9	31.0
2017	230	79	34.4	9	11.4

Note: ^a^The ratio of the number of Acinetobacter baumannii isolates to the number of ICU bacterial isolates

^b^The ratio of the number of CRAB isolates to the number of Acinetobacter baumannii isolates

### Clinical data assessments

Of the 21 cases, 71.42% (15/21) received surgery, and 23.81% (5/21) patients had a tracheostomy or endotracheal tube. 95.23% (20/21) of these isolates were derived from sputum culture. The overall in-hospital mortality was 14.29% (3/21). Twenty-one patients were investigated (median age: 56 range: 25–86 years; males: 61.90%, 13/21) (data not shown). The median hospital stay was 20 days (range 5–65). In particular, antibiotics susceptibility testing for all isolated strains exhibited the same resistance pattern, which is resistance to commonly used antibiotics in the clinic. The clinical details of the patients are presented in Table 2. Patients were treated with antimicrobials including cephalosporins, carbapenems alone or combination with other antibiotics. All improved patients were discharged after treatment or transferred to another hospital, except for the three who died.

**Table 2:** Epidemiological, phenotypic, and genotypic data of *Acinetobacter baumannii* isolates and of patients

Patient	ERIC-PCR	ST	Clinical sample	Hospital stay days	Outcome	Invasive procedure	OXA-23	OXA-24	OXA-51	ISAbal	ISA-23	TEM	ISA-51	ADC	ISA-ADC
AB001	A	2	sputum	11	death	YES	✓	✓	✓	✓	✓	✓		✓	
AB013	A	2	sputum	22	ordinary	YES	✓	✓	✓	✓	✓	✓		✓	
AB020	A	2	sputum	30	ordinary	YES	✓		✓	✓	✓	✓		✓	✓
AB071	A	2	sputum	15	ordinary	YES	✓	✓	✓	✓	✓	✓		✓	
AB033	A	2	sputum	5	ordinary	YES	✓	✓	✓	✓	✓	✓		✓	
AB063	A	2	sputum	65	ordinary	YES	✓	✓	✓					✓	
AB068	A	2	blood	25	ordinary	YES	✓	✓	✓	✓	✓	✓		✓	
AB073	A	2	sputum	13	death	YES	✓		✓	✓	✓	✓		✓	✓
AB031	B	2	sputum	10	ordinary	YES	✓		✓	✓	✓	✓		✓	
AB032	B	2	sputum	17	ordinary	YES	✓		✓	✓	✓	✓		✓	
AB051	B	2	sputum	53	ordinary	YES	✓		✓	✓	✓	✓		✓	✓
AB082	B	2	sputum	12	ordinary	YES	✓		✓	✓	✓	✓		✓	✓
AB111	B	2	sputum	20	ordinary	YES	✓		✓	✓	✓	✓		✓	
AB122	B	2	sputum	56	ordinary	YES	✓		✓	✓	✓	✓		✓	
AB126	B	2	sputum	14	death	YES	✓		✓	✓	✓	✓		✓	✓
AB131	B	2	sputum	26	ordinary	YES	✓		✓	✓	✓	✓		✓	
AB135	B	2	sputum	25	ordinary	YES	✓		✓	✓	✓	✓		✓	
AB136	B	2	sputum	38	ordinary	YES	✓		✓	✓	✓	✓		✓	
AB137	B	2	sputum	15	ordinary	YES	✓		✓	✓	✓	✓		✓	
AB138	B	2	sputum	10	ordinary	YES	✓		✓	✓	✓	✓		✓	
AB125	C	1199	sputum	65	ordinary	YES	✓		✓	✓	✓	✓		✓	✓

Note: ERIC-PCR:enterobacterial repetitive intergenic consensus- polymerase chain reaction; ST:sequence type determined by the Pasteur MLST

### The genotype of carbapenem-resistant *Acinetobacter baumannii*

Clinical isolates were positive for the following genes: *bla*_OXA23_, *bla*_OXA51_, *bla*_OXA24_, *bla*_ADC_, *bla*_TEM_, *ISAba1*, *ISA-23*, and *ISA-ADC*; however, *bla*_OXA58_, *ISA-51*, *bla*_NDM_, *bla*_IMP_, *bla*_KPC_, *bla*_TEM_, *bla*_SHV_, *bla*_VIM_, and *bla*_ACC_ were not detected. These results are shown in Table 2.

### Horizontal transfer of resistance genes

Conjugation experiments indicated that plasmids containing resistance genes were successfully transferred from four CRAB isolates to recipient *E. coli* J53. *bla*_*OXA-23*_, *bla*_*OXA-51*_, and *ISAba1* producing tranconjugants were successful. Among the four strains of *A. baumannii* that were successfully conjugated, three strains (AB13, AB33, and AB63) belonged to type A of ERIC-PCR, and one strain (AB51) was type B. As show in Table 3, the drug resistance gene *bla*_*OXA-23*_ and the insertion sequence *ISAbal* both successfully transferred into recipient *E. coli* J53; however, the *ISAbal*-*bla*_*OXA-23*_ structure was only successful in type B transfer. Attempts to obtain *bla*_*OXA-24*_- producing tranconjugants were unsuccessful.

**Table 3:** Comparison of donors and transconjugant resistance genes

Number of isolates	16sRNA (AB)	ST	ERIC-PCR	β-Lactamase					IS element			
				bla-OXA-51	bla-OXA-23	bla-OXA-24	bla-ADC	bla-TEM	ISAbal	ISAbal- blaOXA-23	ISAbal-blaOXA-51	ISAbal-blaADC
AB13	+	2	A	+	+	+	+	+	+	+	–	–
JC13	–	no	no	+	+	–	–	+	+	–	–	–
AB33	+	2	A	+	+	+	+	+	+	+	–	–
JC33	–	no	no	+	+	–	–	+	+	–	–	–
AB51	+	2	B	+	+	–	+	+	+	+	–	+
JC51	–	no	no	+	+	–	–	–	+	+	–	–
AB63	+	2	A	+	+	+	+	–	+	+	–	–
JC63	–	no	no	+	+	+	–	–	+	–	–	–

Note: AB13; AB33;AB51; AB63 are the donors bacteria, JC13;JC33;JC51;JC63 are the recipients

No:Not executed; ERIC-PCR:enterobacterial repetitive intergenic consensus- polymerase chain reaction; ST:sequence type determined with the Pasteur MLST scheme

### Homology analysis

**Fig. 1:**
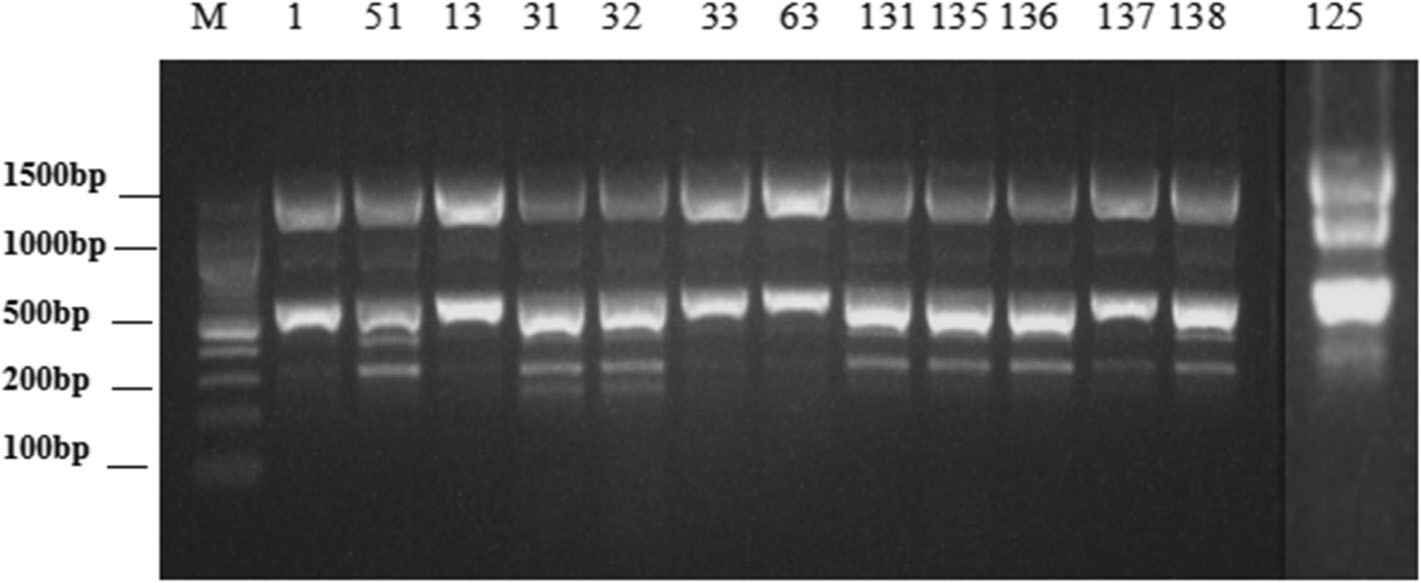
Partial DNA fingerprinting of ERIC-PCR of *Acinetobacter baumannii* strain. Note:M: marker; typeA(1,13,33,63);typeB(51,31,32,131,135,136,137,138);typeC(125)

**Fig. 2:**
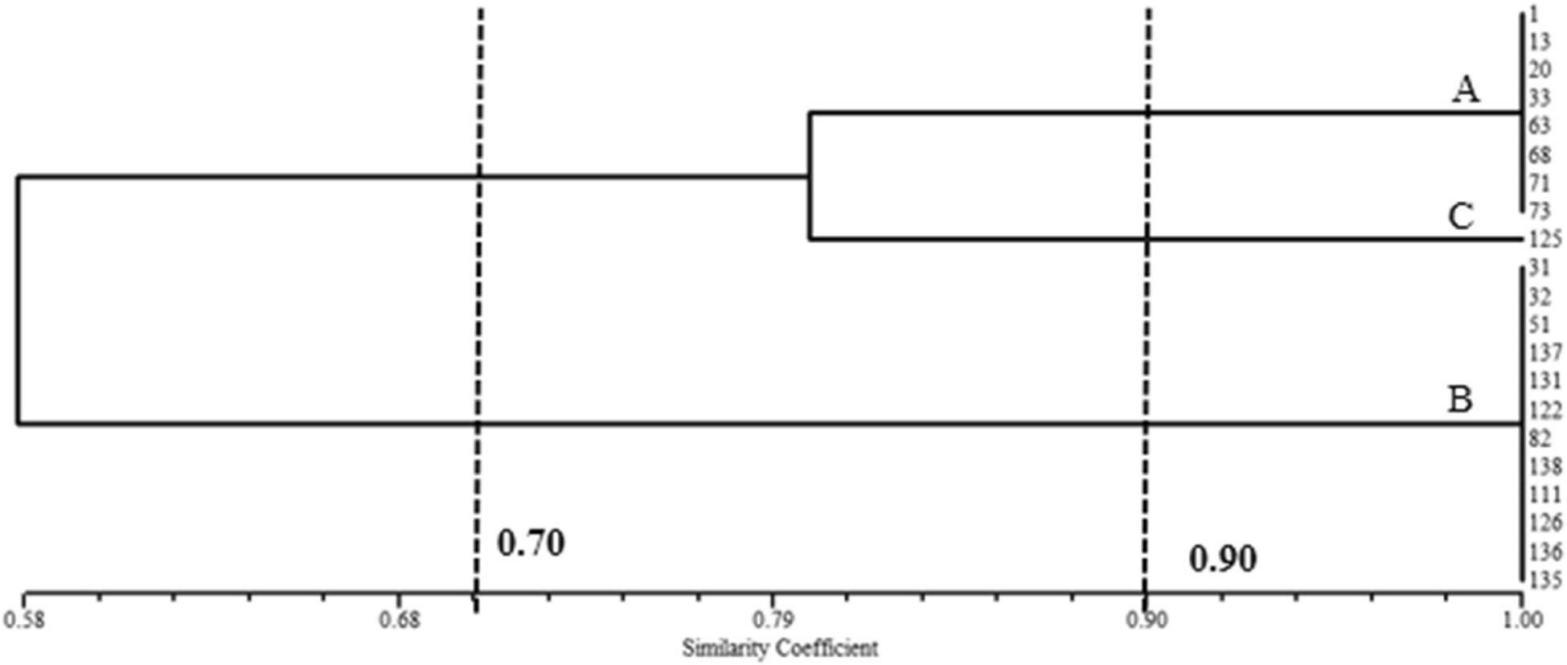
Dendrogram of *Acinetobacter baumannii* strains from ICU samples based on ERIC-PCR analysis. Note: The bottom bar indicates the similarity coefficient, the line on the left indicate the clusters formed in a 70% cut-off. Isolates of more than 90% similarity were treated as a single isolate

MLST showed that all strains corresponded to sequence type 2 (ST2), except for one isolate that belonged to the new genotype ST1199. ERIC-PCR results showed 2–4 bands (Fig. 1) with a size range of 200–1500 bp. Three major groups were identified in the studied *A. baumannii* strains based on the ERIC-PCR banding pattern. Type A in eight, type B in 12, type C in one (Fig. 2), and two profiles (A, B) belonged to ST2.

## Discussion

There have been reports of clonal outbreaks related to environmental reservoirs before [16], nevertheless, the setting in many hospitals is complicated and unpredictable. In these cases, sporadic and epidemic clones, along with different reservoirs such as environmental reservoirs or colonized and infected patients could coexist. Thus, the epidemiology of *A. baumannii* is difficult to explain. An important limitation of the presented research is that environmental strains were not collected from the ICU; therefore, this study lacks surveillance for source identification. Another limitation was the lack of routine culture monitoring for all patients that were newly admitted to the ICU. Therefore, it was impossible to distinguish between new cases which acquired CRAB in the ICU and imported cases which acquired CRAB before admission to the ICU. Yamada et al. reported that invasive procedures, ICU duration, recent surgery, and exposure to broad-spectrum antibiotics are risk factors for colonization or infection by MDR *A. baumannii* [17]. The ICU have been seen as epicenters of the epidemiology of *A. baumannii* [18]. In several previous studies, the unit had to be completely shut down to control the epidemic [19, 20]. However, the ICU in this study could not be closed during hospital outbreak because it was the only referral agency in the area. Furthermore, since there is no single room in the ICU of this hospital, it cannot be completely isolated and disinfected, which also introduces difficulties for infection control. Fortunately, there has been a downward trend since 2015. This outbreak demonstrated that it is effective to implement strict contact precautions and to decrease environmental contamination to control the outbreak of CRAB.

The clinical isolates of *A. baumannii* are mainly composed of three international clone lineages called European clones I, II, and III. In the MLST scheme, most outbreak strains belong to CC1 and CC2, corresponding to European clones I and II, respectively [21]. So far, ST2 type as the most common ST in CC2 has been reported in Australia, Russia, Italy, and China [22–25]. The results of this study show that all strains belonged to ST2 except for one isolate, which belonged to the new genotype ST1199. Furthermore, this study showed that in the clonal isolate, ERIC-PCR was capable to accurately cluster CC2 isolates. Compared to MLST, ERIC-PCR has a cost advantage. These results indicated that MLST combined with ERIC-PCR may be an economical method to solve the hospital epidemiology of *A. baumannii*.

Our results suggest that carrying the *bla*_OXA-23_ gene is the main cause of the CRAB resistance phenotype, which is consistent with previous reports [26, 27]. Interestingly, *bla*_OXA-24_ was also discovered in the presented experiments, and it was also found in Spain, Georgia, France, and the US [28]. This is the first time that *bla*_OXA-24_ has been detected in eastern China. The *bla*_ADC_ was also found in the current study, which is intrinsic in *A. baumannii*. It was found that the insertion sequences(ISs) can activate *bla*_OXA-23_, *bla*_OXA-51_ genes, and *AmpC* [22, 29]. Resistance can arise by mutation and transmission is divided into vertical transmission and horizontal gene transfer (resistance genes are obtained from other bacteria). Intercellular transfer mainly includes three mechanisms: natural transformation (direct DNA transfer), transduction (by phage transfer), and conjugation(by plasmids transfer). Conjugation is the most common drug resistance transmission mode [30]. Therefore, a conjugation experiment was performed to verify the horizontal transfer of drug resistance genes. The *bla*_OXA-51_-like gene is inherent in *A. baumannii* and initially located on chromosomes. However, studies have shown that the *bla*_OXA-51_-like gene and its upstream ISAba1 sequence have been together transmitted via plasmid in Taiwan [31]. In this study, a *bla*_OXA-51_ carrying plasmid was transferred by conjugation to non-*A. baumannii* species (*E. coli* J53). This phenomenon not only further complicates the management of *A. baumannii* infections but also affects the accuracy of *bla*_OXA-51_-like as a standard for distinguishing *A. baumannii*. *bla*_OXA-23_-producing isolates were prevalent in ICU in the First Affiliated Hospital of Jiamusi University hospital, and *bla*_OXA-23_ can be inserted into chromosomes and plasmids and encircled by various genetic contexts. The main reported genetic background includes three categories, including genomic islands, transposons, and ISs [32]. In the present study, all conjugated plasmids contained the *bla*_OXA-23_ gene, but only the type B of ERIC-PCR isolate successfully transferred the *ISAbal*-*bla*_*OXA-23*_ structure in conjugation, indicating that typeA and typeB have different transfer systems and the mechanism needs further verification. The *bla*_OXA-24_ gene found in *A. baumannii* does not have the typical structure associated with DNA mobilization, such as ISs. However, this gene is flanked by XerC/XerD binding sites and may be involved in gene mobilization [32, 33]. This indicates that *bla*_OXA-24_ differs from *bla*_OXA-23_ in both genetic environment and plasmid type. Attempts to obtain *bla*_OXA-24_-producing transconjugants were unsuccessful. This is consistent with previous research [33]. The possible reason is that *bla*_OXA-24_ genes were located in the genome or the plasmid containing the gene cannot be replicated in *E. coli* J53. However, the spread of *bla*_OXA-24_ should still receive attention.

## Conclusions

In summary, this is the first report of the molecular mechanisms and epidemiology of CRAB in eastern China. *bla*_OXA-23_-producing CC2 isolates were prevalent in the ICU of the First Affiliated Hospital of Jiamusi University. Regular survey of molecular epidemiology and health-worker attitudes toward the outbreak of CRAB is important. Targeted investigation to understand the current situation in ICU and implementation of infection control can effectively prevent the spread of nosocomial infection.

## Abbreviations

CCs: Clonal complexes
CHDL: Carbapenem-hydrolysing –lactamases
CRAB: Carbapenem-resistant Acinetobacter baumannii
ERIC-PCR: Enterobacterial repetitive intergenic consensus-PCR
ICU: Intensive care unit
ISs: Insertion sequences
MBL: Metallo-β-lactamases
MHT: Modified hodge test
MICs: Minimum inhibitory concentrations
MLST: Multilocus sequence typing
