# Correction to: Outbreak of carbapenem-resistant Acinetobacter baumannii carrying the carbapenemase OXA-23 in ICU of the eastern Heilongjiang Province, China

**DOI:** 10.1186/s12879-019-4224-8

**Published:** 2019-07-15

**Authors:** Yongxin Zhao, Kewang Hu, Jisheng Zhang, Yuhang Guo, Xuecai Fan, Yong Wang, Sedzro Divine Mensah, Xiaoli Zhang

**Affiliations:** 1 Department of Microbiology, First Affiliated Hospital of Jiamusi University, Jiamusi, Heilongjiang Province China; 2 Second Affiliated Hospital of Jiamusi University, Jiamusi, Heilongjiang China; 3 Jiamusi University, Jiamusi, Heilongjiang China

**Correction to: BMC Infect Dis**

**https://doi.org/10.1186/s12879-019-4073-5**

After publication of the original article [1], we were notified that an author’s name has been incorrectly spelled. Sedzro Divine Mensal should be replaced with Sedzro Divine Mensah.

The original article has been corrected.

The publisher apologies for the inconvenience.
